# Attention to food cues following media multitasking is associated with cross-sectional BMI among adolescents

**DOI:** 10.3389/fpsyg.2022.992450

**Published:** 2022-11-25

**Authors:** John Brand, Delaina Carlson, Grace Ballarino, Reina Kato Lansigan, Jennifer Emond, Diane Gilbert-Diamond

**Affiliations:** ^1^Department of Epidemiology, Geisel School of Medicine at Dartmouth College, Hanover, NH, United States; ^2^Dartmouth Cancer Center, Geisel School of Medicine at Dartmouth College, Hanover, NH, United States; ^3^Department of Biomedical Data Sciences, Geisel School of Medicine, Dartmouth College, Hanover, NH, United States; ^4^Department of Medicine Weight and Wellness Center, Dartmouth Health, Lebanon, NH, United States; ^5^Department of Pediatrics, Geisel School of Medicine at Dartmouth College, Hanover, NH, United States

**Keywords:** multitasking, adolescent, food cue, BMI, attention

## Abstract

**Purpose:**

To measure attention to food cues following a multitask or a sustained attention single task, and further, to examine the associations with current weight status and excess consumption.

**Methods:**

Ninety-six 13-to 17-year-olds were fed a standardized meal and then had their attention to food cues measured following completion of a single sustained attention task, media multitask, or a passive viewing control task. Participants then completed an eating in the absence of hunger paradigm to measure their excess consumption. Adolescents completed each condition on separate visits in randomized order. Attention to food cues was measured by computing eye-tracking measures of attention, first fixation duration, and cumulative fixation duration to distractor images while participants played the video game, Tetris. Participants also had their height and weight measured.

**Results:**

Although not statistically significant, attention to food cues was greatest following a media multitask and weakest following a task that engaged sustained attention when compared to a control. First fixation duration was positively and statistically significantly associated with BMI-Z when measured following a multitask. Cumulative fixation duration was not associated with BMI-Z. There were no associations between BMI-Z and attention to food cues after the attention or control task, nor any association between attention to food cues and eating in the absence of hunger.

**Conclusion:**

Among adolescents, we found that current adiposity was related to attention to food cues following a multitask. Multitasking may perturb the cognitive system to increase attention to food cues.

## Introduction

Childhood is a critical period in obesity prevention. Children are learning lifelong eating behaviors, and obesity in childhood often tracks into adulthood leading to an increased risk for health problems including heart disease, type-2 diabetes, and several cancers (Clinical guidelines on the identification, evaluation, and treatment of overweight and obesity in adults: executive summary, 1998). Understanding individual factors that lead some children to overconsume, and ultimately gain excess weight, is essential to the development of effective obesity prevention and treatment strategies (Peirson et al., 2015). A prominent obesity risk factor for the development of obesity may be increased cue reactivity to food cues. It is well established that homeostatic systems regulate feeding to meet energy needs, but the dopaminergic mesolimbic reward system in the brain also drives hedonic consumption leading to excessive caloric intake (Guyenet and Schwartz, 2012). Environmental food cues, such as the sight of food, can induce cravings and lead to heighted food cue reactivity in the reward pathways (Volkow et al., 2011). Higher levels of food cue reactivity measured *via* functional magnetic resonance imaging (fMRI) have been shown to be associated with higher subsequent snack food consumption and adiposity gain among adults and children (Boswell and Kober, 2016; Gilbert-Diamond et al., 2017; Rapuano et al., 2017).

The amount of attention given to palatable food cues may be used as a proxy measure for food cue reactivity in children and adolescents (Werthmann et al., 2011; Brand et al., 2020). Attention to food cues develops *via* an incentive salience feedback loop in which palatable food cues stimulate neural reward activity and prompt individuals to have greater cravings for and seek out energy-dense, nutrient-poor foods in their environment. Through repeated exposure, cues associated with palatable food intake become increasingly salient, resulting in heighted saliency of food cues in the environment (Castellanos et al., 2009).

Attention to food cue research in adults (> 18-years-of-age) and children (< 12 years) shows a positive association with increased weight and consumption.(Castellanos et al., 2009; Werthmann et al., 2011; Folkvord et al., 2015; Brand et al., 2020; Folkvord et al., 2020) The association between the amount of attention given to food cues and weight and consumption among adolescent is not well explicated. Adolescents may be at heightened risk for increased attention to food cues because the adolescent brain is in a sensitive period development (Fuhrmann et al., 2015; Daly et al., 2022) when reward seeking behaviors associated with cue reactivity may have particularly detrimental outcomes, disrupting brain development and increasing the likelihood that maladaptive behaviors persist into adulthood (Crews et al., 2007; Fuhrmann et al., 2015). However, attention to food cue studies among adolescents has been limited to comparisons of BMI between a special population and healthy weight controls, with mixed results. Attention to food cues has been associated with lower BMI among females with binge eating disorder(Schmidt et al., 2016) and positively associated with BMI-Z among adolescents with loss of control eating(Shank et al., 2015) and those with body image concerns (Yokum et al., 2011). A final study found that adolescents with or without anorexia nervosa demonstrated similar levels of attention to food cues (Werthmann et al., 2019), Understanding this relationship in a healthy weight population may help to identify those at high risk for excess weight gain at a critical time in development.

The time course of attentional bias may be measured by monitoring eye movements (Mazidi et al., 2019). Eye-tracking has good spatial and temporal precision, allowing researchers to isolate discrete attentional bias measures that reflect both the early and late aspects of attention. An example of an early attention to food cues measure is first fixation duration, which measures the strength of a stimulus to initially attract and hold one’s attention after it has been initially fixated upon. The initial fixation occurs rapidly suggesting that is driven by bottom-up processes, involving an automatic/involuntary response to look at the stimulus because of its properties (Pinto et al., 2013). Conversely, cumulative gaze duration is an example of a later measure of attention to food cues that measures the strength of a stimulus to hold one’s attention for a sustained period. Its measurement over time suggests that is influenced by top-down goal-oriented processes that are under the conscious control of the participant (Pinto et al., 2013). Compared to conscious measures, automatic attention measures to food cues have been shown to have more robust associations with cross-sectional weight status (Brand et al., 2020), weight loss (Werthmann et al., 2015), and consumption among children (Folkvord et al., 2015).

The modern obesogenic environment is replete with cues to eat, and much research has focused on the role of the obesogenic environment as a risk factor for the development of heightened attention to food cues. Marketing has targeted children by tailoring food advertisements to increase appeal (Bernhardt et al., 2013; Cairns et al., 2013; Castronuovo et al., 2021) and exposure to advertisements has been positively linked to increased weight (Cairns et al., 2013; McClure et al., 2013), as well as excess consumption measured by eating in the absence of hunger (EAH) paradigms (Gilbert-Diamond et al., 2017), a predictor of current and future weight status. Media multitasking in particular—defined as simultaneously engaging in multiple forms of media—has received particular interest because it may widen the “attentional spotlight” leading individuals to be more distracted to external environmental cues (Cain and Mitroff, 2011; Brand et al., 2022), particularly among adolescents who demonstrate high media multitasking behaviors. Youth are spending more time than ever using mobile devices and social media. Youth daily media use has grown faster during the COVID-19 pandemic than the combined 4 years prior to the pandemic (Rideout et al., 2021). From 2019 to 2021, daily use increased to 8 h and 39 min from 7 h and 22 min among 13-18-years-old. It has been reported that adolescents multitask between 40% and 60% of the time they were using media (Limtrakul et al., 2018; Xu et al., 2019).

High levels of media multitasking have been associated with increased adiposity in college-age (Lopez et al., 2020) and pre-adolescents(Lopez et al., 2019) as well as increased cue reactivity to hedonic food stimuli (Lopez et al., 2020). Given the high prevalence of food ad exposure, it may be that multitasking results in a heighted response to food cues in the environment (Crews et al., 2007), thereby increasing hedonically driven consumption and weight gain. In addition, adolescents with overweight or obesity may be at greatest risk for a multitasking-related increase in attention to food cues, because those food cues may be particularly salient to them. In contrast to multitasking, single tasking, defined as completing a singlet task that requires sustained attention, may narrow the attentional spotlight, resulting in less attention to task-irrelevant cues in the environment. Thus far, the effects of multitasking and single tasking on attention to food cues and cued eating have not been examined, nor have differences by weight been evaluated. The present work aims to address this gap by examining these relationships among adolescents, for which the association between media multitasking and attention to food cues may be most pronounced. To accomplish this, we conducted a secondary data analysis of a data set collected from a larger project looking at the association between media multitasking and attention among adolescents (Brand et al., 2022).

### Purpose of the present work

Our primary aim is to quantify differences in measures of attention to food cues following a multitask or a sustained attention single task, and further, to examine the associations with current weight status and acute excess snack consumption measured using the EAH paradigm. We had two primary hypotheses. The first was that compared to a baseline control, attention to food cues will be higher immediately following a multitask and lower following a sustained attention task. The second was that there would be positive associations between attention to food cues and the outcomes of (1) weight status and (2) snack consumption, and that these associations would be strongest in the multitasking condition and weakest in the sustained condition.

## Materials and methods

### Participants

Ninety-six adolescents aged 13–17 years were recruited from rural New Hampshire and Vermont using fliers, community Listservs, social media, and community events. Eligibility criteria included English fluency and absence of health conditions or medication use that may impact appetite or attention span. Of the 96 adolescents who were enrolled, 3 dropped out after visit one, and a further 7 were excluded because of poor eye-tracking calibration in at least one of the visits. Thus, a total of 86 participants with complete data were included in the final analyses.

### Study overview

Participants made three separate visits to the laboratory, approximately 1-week apart. During each visit, adolescents first ate a standardized pre-load meal to satiety and then completed an acute media multitask, sustained attention task, or passive viewing control, randomized by laboratory visit. Immediately after, we then measured attention to food cues by monitoring eye movements to food images while adolescents played the video game, Tetris (Brand et al., 2022). Adolescents were then provided snack foods to eat *ad libitum* to measure EAH. Adolescents also reported on their demographics, and their height and weight were measured at the first visit. All study procedures were approved by the Committee for the Protection of Human Subjects at Dartmouth College. Adolescents provided assent, and parents provided consent.

### Standardized pre-load meal

At the start of each visit, children were offered a hearty pre-load snack that was designed to provide 27% of their daily estimated energy requirement so that they would eat to satiety. The Institute of Medicine’s Estimated Energy Requirement Calculator^1^ was consulted to compute the estimated energy requirement for each child based on their sex, age, height, and weight. The snack consisted of whole grain crackers (18% of snack kcals), cheddar cheese slices (20% of snack kcals), apple slices (19% of snack kcals), peanut butter (26% of snack kcals), carrot sticks (3.4% of snack kcals), a cup of 2% milk (13% of snack kcals), and a cup of water. Children were free to ask for seconds of any of the snack items, in which case 1.5 times the weight of the first serving was provided. They were given up to 20 min to eat *ad libitum*. An additional five additional minutes were offered if the child asked for more time at the end of the first 20 min. Foods were pre- and post-weighed, and nutrient labels and the USDA National Nutrient Database for Standard Reference were consulted to compute caloric consumption.

### Multitask manipulation

#### Stimuli and apparatus

There were three conditions: (1) acute media multitask; (2) sustained attention; and (3) passive viewing control condition as described previously (Brand et al., 2022). In each condition, the screen was divided down the center. The left side of the screen presented yellow and blue dots that measured 4.0° of visual angle when viewed at 60 cm. The location of the dots was randomized to appear within the boundaries of the left side. The right side of the screen contained an image of a mobile phone that displayed messages to the participant using an automated messaging bot.

#### Sustained attention condition

The Go/No-go task was used as our sustained attention task. A blue or yellow dot briefly appeared for between 20 and 1,500 ms and then disappeared. Participants were instructed to press the space bar as quickly as possible to the presence of a blue dot (Go trials) and told not to respond when presented with a yellow dot (No-go trials). At random intervals between 2000 and 5,000 ms, the messaging bot would ding and send participants a text message. Participants were instructed to ignore the competing text messages and focus on the Go/No-go task. Participants completed 100 Go trials and 100 No-go trials randomized on a trial-to-trial basis. There were 60 messages sent to participants throughout the trials.

#### Acute media multitask condition

The multitask condition was identical to the sustained attention condition, with the exception that participants were asked to attend to and type answers to the text messages. Participants were instructed to guess if they did not know the answer. Participants completed 200 trials and received a total of 60 messages.

#### Passive viewing baseline condition

Participants were asked to passively watch a video simulation of the media multitask condition with no specific instructions of where to look.

### Measuring attention to food cues

#### Tetris

Attention to food cues was measured while participants had their eye movements tracked playing the video game Tetris. Binocular eye movements were recorded at 1000 Hz using the Eyelink 1,000 (SR research, Mississauga, ON, Canada). The Tetris game was presented inside a 768 × 1,366 rectangle presented in the middle of the screen. The game was bordered on the left and right by two gray rectangles, measuring 768 × 200 pixels. Distractor images were displayed in the center of the rectangles, measuring 200 × 200 pixels. One image was a picture of a palatable food and the other was a picture of an animal. The food and animal images were taken from the food-pics database (Blechert et al., 2014). The location of the images was randomized, and each image was replaced after 20 s. Total game play time was 5 min.

### Eating in the absence of hunger

Children were left alone in an observation room for approximately 20 min to watch a television show (Mythbusters, Beyond television productions) and were provided with a snack (gummy bears, 240 g ± 1 g; goldfish crackers, 76 g ± 0.5 g; and grapes, 235 g ±1) and water that they could eat *ad libitum*. Gummy bears and goldfish crackers were chosen because they have been previously used as palatable snacks in previous EAH studies (Gilbert-Diamond et al., 2017). Grapes were chosen as a palatable healthy food.

### Anthropometrics

Adolescent weight and height were measured using a Seca 763 Medical Scale and Seca 213 Stadiometer (Hamburg, Germany), respectively. The measurements were used to compute age- and sex-adjusted BMI z-scores using the United States Center for Disease Control and Prevention 2000 growth charts (Kuczmarski et al., 2000).

### Demographics

Parents provided their child’s age, sex, race, and ethnicity. Parents also reported their household income and their highest educational level completed.

### Dependent variables

#### Attention to food cues

Eye movement measures of attention to food distractors were calculated for each participant in each condition (sustained attention condition, multitask, and passive control). Initial gaze duration was measured as the average amount of time the adolescent spent fixating on a food distractor the first time that they looked at it. Cumulative gaze duration was calculated as the total amount of time participants spent fixating on food distractors, summed over all fixations. Areas of interest were created around the food and animal images, defined as the area of the image, 200 × 200 pixels. Attention measures were measured in milliseconds.

### Statistical analysis

R language and environment for statistical computing was used to compute all analyses. Distributions of each attention to food cue measure and BMI-z scores were compared across parent, child, and household characteristics using unadjusted linear regression. Any variables which were associated (*p* < 0.10) with any outcome variables were included as part of any adjusted analyses. For all analyses, we used a statistical significance threshold of *p* < 0.05.

Separate mixed-effects regressions were used to assess whether attention to food cues increased following acute media multitasking and decreased following the single sustained attention task relative to the passive viewing baseline. First fixation duration and cumulative fixation duration were predicted from condition, coded as binary (media multitask = 1 vs. passive viewing control = 0; or, separately, sustained attention = 1 vs. passive viewing control = 0); a random effect was included at the participant level to account for repeated measures within participant. Mixed-effects linear regressions were also used to evaluate the associations between attention to food cues with BMI-Z scores and EAH. BMI-Z was predicted from each attention to food cues metric (first fixation duration or cumulative fixation duration, separately) with study condition coded as binary (media multitask = 1 vs. passive viewing control = 0; or, separately, sustained attention = 1 vs. passive viewing control = 0), and an interaction term between the attention metric and study condition. We then repeated those mixed-effects linear regressions using EAH as the outcome. All regressions were repeated adjusting for sex, age, and the percent expected energy requirement consumed at the pre-load meal.

## Results

**Figure 1:**
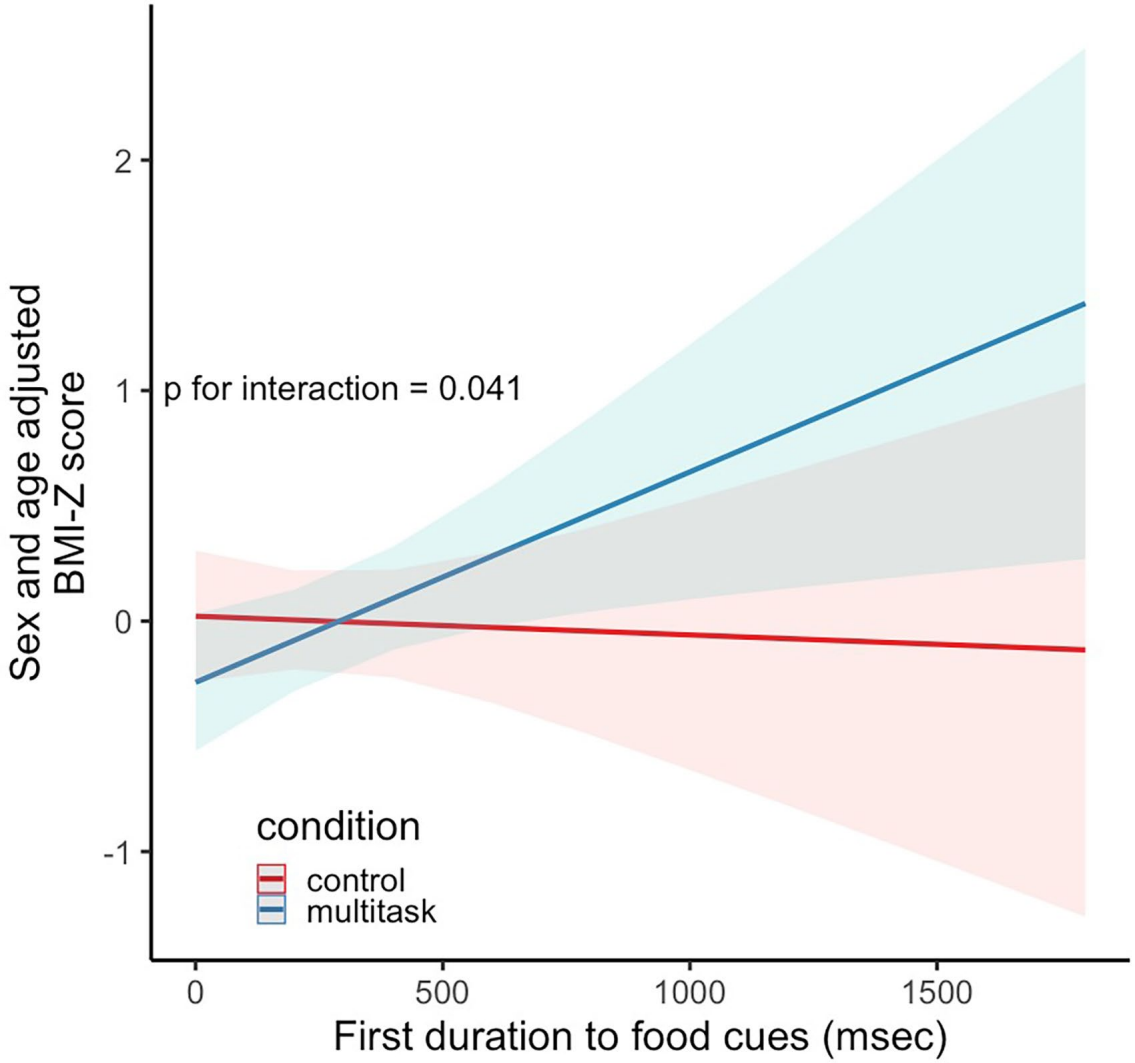
Predicted sex and age adjusted BMI-Z scores for the first fixation to food cues by condition (multitask versus control) interaction. Shaded areas represent the standard error.

There were no significant associations in the distributions of attention to food cues metrics and BMI-Z scores across parent, child, and household characteristics. Distributions are presented in the Supplementary material. First fixation duration and cumulative fixation duration increased after the acute media multitasking condition and decreased after the sustained attention task condition compared to after the passive viewing task condition, though the differences were not statistically significant (Table 1). When examining the association between first fixation duration on BMI-Z scores following completion of the multitask vs. control conditions, there was a statistically significant interaction (*p* = 0.041). Visual inspection revealed a positive association between BMI-Z score and first fixation when measured following completion of the media multitask. There was no association between BMI-Z score and first fixation following completion of the control task (Figure 1). There was no significant interaction between cumulative fixation duration and condition following completion of the sustained attention versus control conditions (*p* = 0.533). There were also no observed statistically significant associations between attentional metrics and EAH in any of the conditions. The results remained unchanged after adjusting for age, sex, and the percent expected energy requirement consumed at the pre-load meal.

**Table 1:** Unadjusted linear models predicting first fixation duration and cumulative fixation duration from condition.

	First fixation duration		Cumulative fixation duration	
	Mean (SD)	*p* *	Mean (SD)	*p*
CONDITION				
Passive viewing control	Reference	--	Reference	--
Multitask	25.2	0.598	505	0.84
Sustained attention	−33.9	0.478	−872	0.73

* p-values calculated from linear regressions predicting first fixation or cumulative fixation from multitask or sustained attention condition versus control.

## Discussion

In the present work, we assessed measurements of attention to food cues following multitasking, sustained attention, and passive viewing tasks and whether they were associated with BMI-Z scores and EAH. Our results suggest that tasks completed immediately prior may affect attention to food cue measurements. Qualitatively, attention to food cues was greatest following a media multitask and weakest following a task that engages sustained attention, although attention metrics in the media multitask and sustained attention task were not statistically significantly different than the passive viewing control. We also found that first fixation duration was positively associated with BMI-Z when measured following a multitask, but not when measured following a sustained attention task relative to viewing a control. There were no observed associations between attention to food cues and EAH.

Overall, our results corroborate research suggesting media multitasking may be a risk factor for the development of obesity, demonstrating an association between attention to food cues and current obesity following an acute multitask. Completing multitasks has been shown to drain attentional resources, specifically related to the ability to focus attention and filter irrelevant information (Ophir et al., 2009; Cain and Mitroff, 2011). The present results may also be understood within the ego depletion framework of self-regulation(Baumeister et al., 1998) that attempts to explain why some people are predisposed to fail at cognitive tasks (Baumeister and Vohs, 2007). It postulates that exercising self-control—such as dividing attention between two tasks—depletes cognitive resources affecting performance. It may be that completing the multitask in our study required more self-control (to maintain both tasks in parallel) than completing the control condition, which did not require the completion of a specific task. Inhibiting attention to food distractors after the multitask condition would thus be harder when compared to the control condition.

We did not observe an association between attention to food cues and measured consumption in an EAH paradigm. Food stimuli in our study was not associated with the foods presented in the EAH paradigm. This contrasts a previous eye-tracking study showing an association between attention to food cues and consumption among children, but only for foods that were used in the measurement of attention to food cues (Folkvord et al., 2015). That study also did not provide a pre-load meal so it possible that the children had elevated attention to food cues that led to increased consumption. Because children in our study first ate to satiety, this may have led to a decreased relationship between attention to food cues and cued eating. It is important for future research to determine under which conditions (e g., fasted vs. fed) attention to food cues is most strongly associated with excess consumption.

The present results also contrast research that use attention to food cues as a behavioral measure of the temperament trait, appetite arousal that measures the emergence of the desire to eat. A study of 7-12-year-olds showed that increased appetite arousal was associated with decreased self-regulation while eating in the absence of hunger (i e., increased consumption), but not associated with BMI (Godefroy et al., 2017). Rather, BMI was associated with the temperament trait, appetitive persistence (i.e., the continuing desire to eat). This discrepancy in results may be explained by the age difference between our samples and the fact that this study measured attention to food cues indirectly using reaction time. Previous studies have demonstrated that early measures of attention are more robustly associated with current weight status rather than later measures (Werthmann et al., 2015; Brand et al., 2020). Indirect measures are limited because they cannot differentiate early attentional processes from the later attentional stages. Additionally, because our participants were older, they may have had increased inhibitory control that affected their EAH. Inhibitory control increases within the adolescent period^41^and adolescents exhibit better inhibitory control than children, and similar levels to emerging adults (Williams et al., 1999).

To our knowledge, the present work is the first to experimentally measure the effects of multitasking on attention to food cues and cued eating. Our finding of a positive association between attention to food cues and current weight status only after completing a multitask provides support for the hypothesis that media multitasking impacts attention to make children become more responsive to food cues in their environment. As such, our work may have important implications for researchers who wish to use attention to food cue measures to study obesity. It may be beneficial to measure such metrics following tasks—such as multitasking—that perturbs a participant’s cognition when the association with obesity is strongest. Having participants complete the same task prior to an attentional measurement may place all participants in the same state, preventing outside factors from influencing collected data.

The present work must be understood with respect to the following limitations. We attempted to develop a novel multitask to experimentally investigate the effects media multitasking has on attention to food cues. However, as described elsewhere (Brand et al., 2022), our ability to interpret the results is limited because of our inability to measure task engagement with our multitask. It is important for future research to be able to measure task engagement accurately to ensure robust data collection and adequate controls, such as controlling for variability in task difficulty among participants and confirming that participants are completing the task as designed. The present work also did not measure temperamental traits, which are known to play a critical role in the ability of children and adolescents to self-regulate food intake and their weight status. Inhibitory control is an executive functioning process ( Posner and Rothbart, 2000) that has been studied extensively in relation to eating behaviors. Lower inhibitory control has been linked with binge eating behaviors among adolescents (Ames et al., 2014; Kittel et al., 2017), lower abilities to self-regulate intake (Tan and Holub, 2011), increases in food enjoyment and food responsiveness (Groppe and Elsner, 2015), and higher weight (Nederkoorn et al., 2006, 2012; Houben et al., 2014). Understanding temperamental traits may help to explain why adolescents with higher weight status may be particularly prone to the effects of multitasking on attention to food: It may exacerbate their already weaker general and food-specific inhibition, and weaker executive function (Dassen et al., 2018).

## Conclusion

In general, these results show that tasks completed immediately prior may affect measurements of attention to food cues. Among adolescents, we found that current adiposity was related to attention to food cues following a multitask, suggesting that engaging in multitasking may perturb the cognitive system to increase attention to food cues.

## Data availability statement

The raw data supporting the conclusions of this article will be made available by the authors, without undue reservation.

## Ethics statement

The studies involving human participants were reviewed and approved by the Committee for the Protection of Human Subjects at Dartmouth College. Written informed consent to participate in this study was provided by the participants’ legal guardian/next of kin.

## Author contributions

JB, JE, and DG-D contributed to the conception and design of the study. JB performed the statistical analysis and wrote the first draft of the manuscript. DC, GB, JE, and RL contributed to specific sections of the manuscript. All authors contributed to the article and approved the submitted version.

## Funding

This work was supported by grants R01HD092604 and R21HD097475 from the Eunice Kennedy Shriver National Institute of Child Health and Human Development.

## Conflict of interest

The authors declare that the research was conducted in the absence of any commercial or financial relationships that could be construed as a potential conflict of interest.

## Publisher’s note

All claims expressed in this article are solely those of the authors and do not necessarily represent those of their affiliated organizations, or those of the publisher, the editors and the reviewers. Any product that may be evaluated in this article, or claim that may be made by its manufacturer, is not guaranteed or endorsed by the publisher.

## Supplementary material

The Supplementary material for this article can be found online at: https://www.frontiersin.org/articles/10.3389/fpsyg.2022.992450/full#supplementary-material

## Footnotes

^1^ https://globalrph.com/medcalcs/estimated-energy-requirement-eer-equation/
