# Supplementary material for: Attention to food cues following media multitasking is associated with cross-sectional BMI among adolescents
Source: Front Psychol. 2022 Nov 25;13:992450. doi: 10.3389/fpsyg.2022.992450 (PMC9732437; doi:10.3389/fpsyg.2022.992450)
Supplement: Supplementary file 1 [file Table_1.DOCX]

| **Supplementary Table 1.** Distribution of attention to food metrics in the passive viewing control condition and BMI-Z scores across child, parent, and household characteristics. | | | | | | |  |
| --- | --- | --- | --- | --- | --- | --- | --- |
|  |  |  | First fixation duration  (msec) |  | Cumulative fixation duration  (msec) | BMI Z-score |  |
|  | N |  | Mean (SD) |  | Mean (SD) | Mean (SD) |  |
| **Overall** | 86 |  |  |  |  |  |  |
| **Child Characteristics** |  |  |  |  |  |  |  |
| Age, years |  |  |  |  |  |  |  |
| 13 | 25 |  | 234.80 (225.02) |  | 11262.32 (19949.38) | 0.126 (1.19) |  |
| 14 | 20 |  | 269.40 (307.63) |  | 7466.25 (12888.42) | -0.29 (0.91) |  |
| 15 | 24 |  | 290.75 (335.19) |  | 10710.13 (15256.03) | 0.17 (1.01) |  |
| 16 | 14 |  | 232.07 (286.39) |  | 7356.21 (10829.20) | 0.04 (0.67) |  |
| 17 | 3 |  | 219.67 (312.96) |  | 3084.33 (2948.07) | -0.68 (0.79) |  |
| β (95% CI) |  |  | 2.68 (-50.08, 55.44) |  | -1075 (-3902.13, 1751.53) | -0.033 (-0.22, 0.15) |  |
| Sex |  |  |  |  |  |  |  |
| Male | 47 |  | 279.15 (304.70) |  | 11257.00 (18629.37) | 0.02 (1.12) |  |
| Female | 39 |  | 231.39 (261.36) |  | 6950.95 (9764.39) | -0.02 (0.84) |  |
| β (95% CI) |  |  | -47.76 (-170.92, 75.39) |  | -4306 (-10883.65, 2271.55) | -0.54 (-0.49, 0.38) |  |
| Race |  |  |  |  |  |  |  |
| White, non-Hispanic | 71 |  | 259.08 (355.83) |  | 10282.92 (14768.13) | -0.46 (0.55) |  |
| Other, non-Hispanic | 12 |  | 248.76 (243.19) |  | 9432.20 (15762.93) | 0.11 (1.04) |  |
| β (95% CI) |  |  | 34.24 (-33.46, 101.94) |  | 1861.60 (-1787.15, 5510.31) | 0.14 (-0.99, 0.37) |  |
| **Parent Characteristics** |  |  |  |  |  |  |  |
| Mother’s education level |  |  |  |  |  |  |  |
| High school diploma | 2 |  | 86.00 (83.44) |  | 4518.50 (1790.62) | 0.04 (1.22) |  |
| Associate or Bachelor’s degree | 69 |  | 266.17 (178.44) |  | 5390.75 (8871.12) | 0.28 (1.04) |  |
| Graduate school | 12 |  | 252.15 (274.38) |  | 9266.01 (14138.36) | -0.02 (0.99) |  |
| β (95% CI) |  |  | 15.28 (-34.11, 64.66) |  | 1591 (-4243.32, 1061.16) | -0.09 (-0.269, 0.072) |  |
| **Household Characteristics** |  |  |  |  |  |  |  |
| Annual income |  |  |  |  |  |  |  |
| $0-64, 999 | 4 |  | 151.25 (186.87) |  | 2559.50 (4458.43) | -0.38 (1.14) |  |
| $65,000-144,999 | 64 |  | 253.19 (265.71) |  | 8612.52 (13761.01) | 0.04 (0.96) |  |
| > $145,000 | 12 |  | 202.67 (200.21) |  | 14204.17 (23060.12) | -0.01 (1.27) |  |
| β (95% CI) |  |  | 26.07 (-39.33, 91.47) |  | 1674 (-1854.44, 5202.90) | -0.05 (-0.283, 0.184) |  |
| **Note:** Beta and 95% confidence intervals around the beta were calculated from linear regression models with the participant characteristic as the single exposure and either the attention metric or usual media multitasking as the continuous outcome. Age coded as continuous. Sex and race coded as binary. Mother’s education level coded as ordinal. 0 = some high school, 1 = High school graduate or GED; 2 = Associates degree, 3 = Bachelor’s degree, 4 = Graduate school (including medical and law school). Household income coded as ordinal: 0 = less than $25, 000, 1 = $25, 000-64,999, 2 = $65,000-144,999, 3 = $145, 000-224,999, 4 = 225,000 or more. | | | | | | | |
